# Anesthetic management for inhibiting sympathetic activation in an adolescent patient diagnosed with catecholaminergic polymorphic ventricular tachycardia and undergoing left cardiac sympathetic denervation: A case report

**DOI:** 10.1002/ccr3.7658

**Published:** 2023-07-02

**Authors:** Kyung‐Hwa Kwak, Young‐Woo Do, Taeyoung Yu, Jinyoung Oh, Sung‐Hye Byun

**Affiliations:** ^1^ Department of Anesthesiology and Pain Medicine, Kyungpook National University Chilgok Hospital, School of Medicine Kyungpook National University Daegu South Korea; ^2^ Department of Thoracic and Cardiovascular Surgery, Kyungpook National University Chilgok Hospital, School of Medicine Kyungpook National University Daegu South Korea

**Keywords:** case report, catecholaminergic polymorphic ventricular tachycardia, nerve block, perioperative care, sympathectomy

## Abstract

Catecholaminergic polymorphic ventricular tachycardia (CPVT) is a genetic disorder in which catecholamine release during exercise or emotional stress cause fatal tachyarrhythmias. In this paper, we discuss methods to minimize the sympathetic stimulation that can occur during the perioperative period in patients undergoing left cardiac sympathetic denervation to surgically treat CPVT.

## 1 INTRODUCTION

Catecholaminergic polymorphic ventricular tachycardia (CPVT) is an inherited arrhythmia syndrome in which catecholamine release induced by exercise or emotional stress causes fatal tachyarrhythmia such as bidirectional or polymorphic ventricular tachycardia (VT).^1^, ^2^, ^3^ As a physiological fight‐or‐flight response under stress conditions, the released catecholamine activates beta‐adrenergic receptor signaling to enhance the movement of calcium load into the cardiac cell and calcium uptake into the cardiac sarcoplasmic reticulum (SR).^4^ However, if a CPVT‐related gene mutation exists, the cardiac ryanodine receptor (RyR2), a calcium‐release channel located in the cardiac SR, spontaneously opens during diastole. This action causes an unregulated pathological calcium release, thereby resulting in delayed after‐depolarization (DAD), which leads to triggered beats that induce ventricular arrhythmia.^5^ Therefore, in patients with CPVT, sympathetic activation should be suppressed. Even when CPVT is diagnosed and a patient undergoes left cardiac sympathetic denervation (LCSD) as treatment, methods are needed to minimize sympathetic stimulation that can occur during the perioperative period. Hence, we discuss a method for the smooth induction, maintenance, and emergence of anesthesia in CPVT patients undergoing LCSD.

## 2 CASE REPORT

**FIGURE 1:**
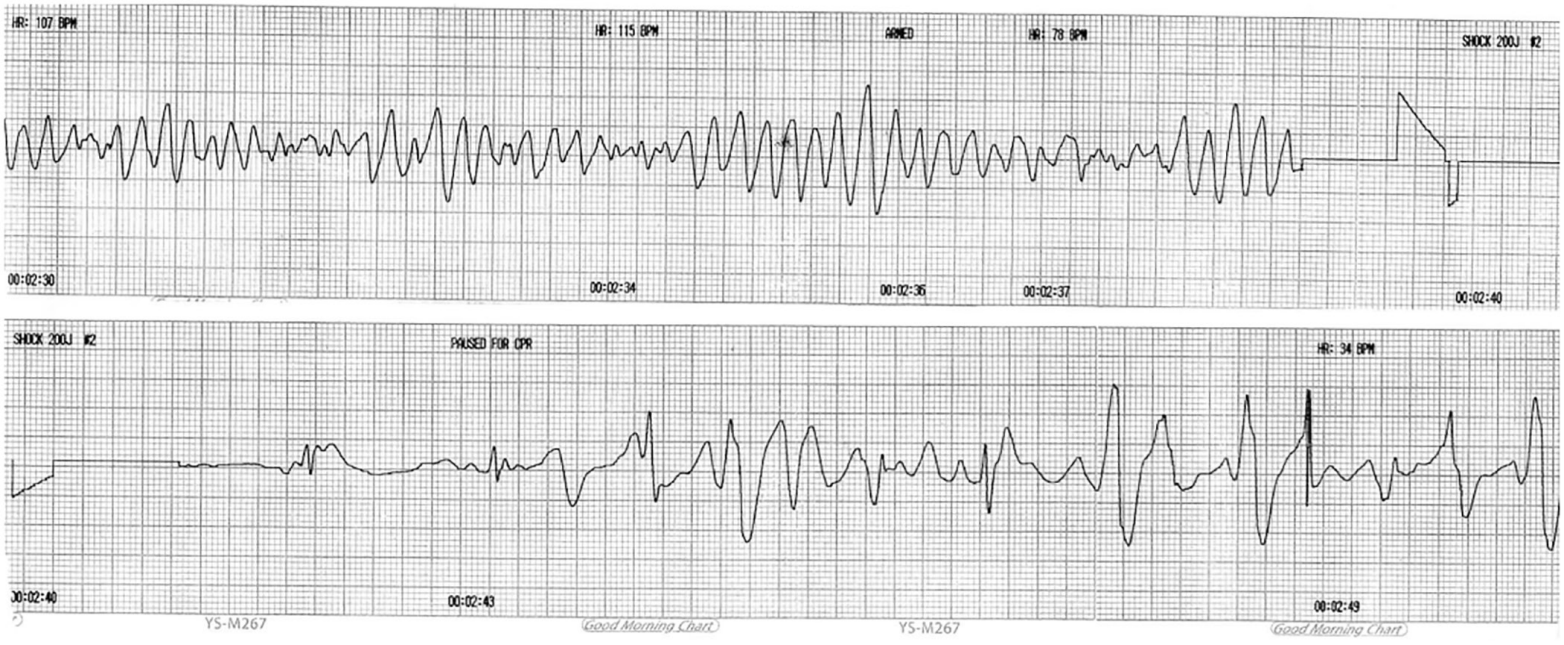
The defibrillator strips show resuscitation from ventricular fibrillation when shocked with 200 J.

A 13‐year‐old girl (height, 170 cm; weight, 50 kg) was diagnosed with CPVT and LCSD was planned. She had a history of loss of consciousness 3 years earlier and has not participated in physical activities during school physical education classes because of frequent syncope events when climbing more than three flights of stairs or while exercising. Two months earlier, she had a sudden cardiac arrest due to ventricular fibrillation (VF) at school, and spontaneous circulation was returned after a defibrillator was used three times (Figure 1). At that time, she was transferred to our hospital for further management. While admitted to the pediatric intensive care unit (PICU), antiarrhythmic medications, including amiodarone and bisoprolol, were administered. CPVT or long QT syndrome (LQTS) was suspected, based on the past and present history; therefore, a next‐generation sequencing panel was performed. An *RYR2* mutation was subsequently identified through genetic testing. She was referred to the department of thoracic surgery to undergo LCSD, given the possibility of future recurrence of tachyarrhythmia, although she had no related symptoms after starting the antiarrhythmic medication. Preoperative electrocardiography conducted in the PICU revealed sinus bradycardia with a heart rate (HR) of 40–50 bpm, which is thought to be an effect of the anti‐arrhythmic agents being taken. At the preoperative visit, the patient and her parents were given detailed information on the anesthetic management in CPVT patients, including precautions to be taken in them and the procedures to be performed during anesthesia by an anesthesiologist.

**FIGURE 2:**
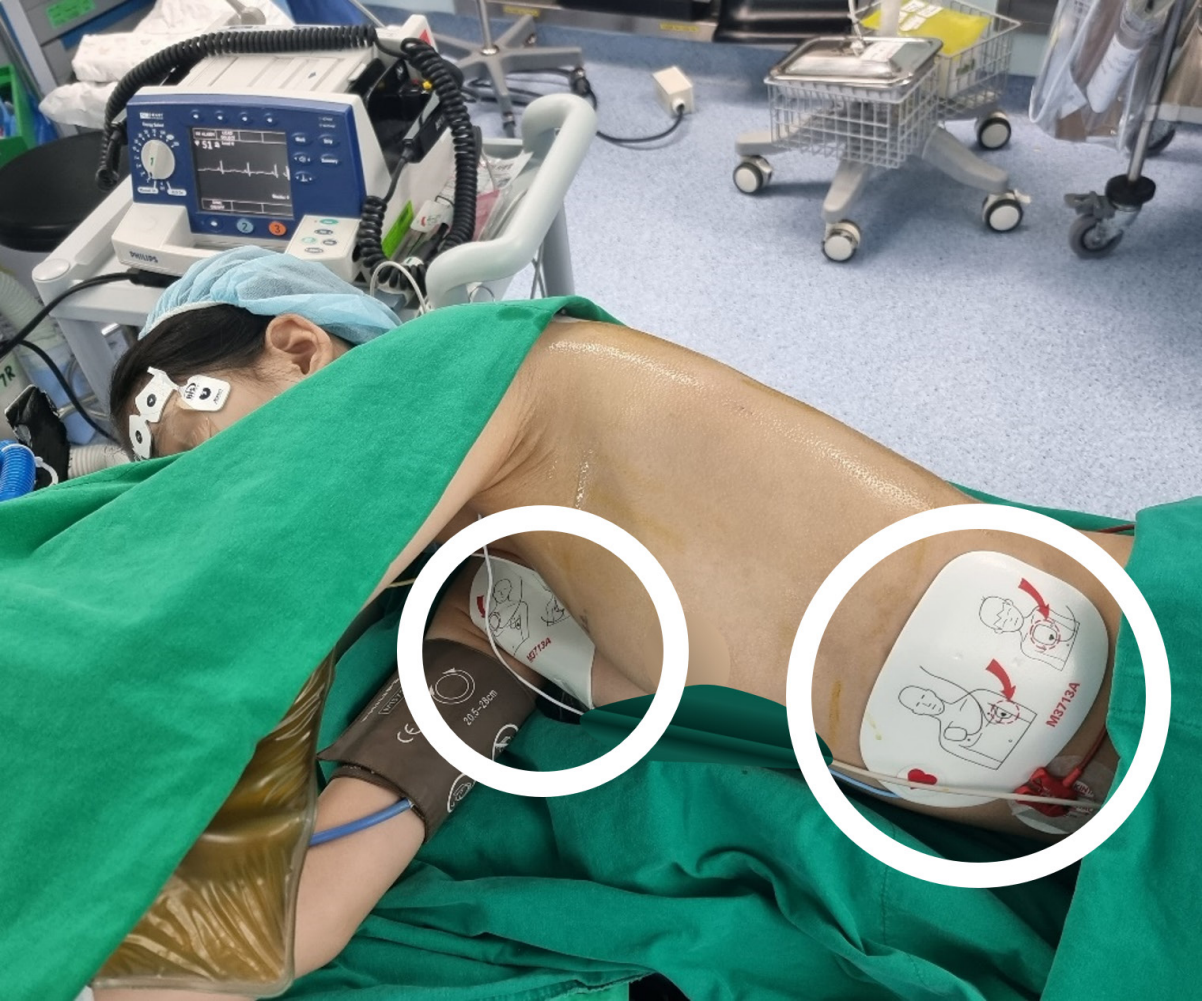
Setting the defibrillator for unexpected intraoperative ventricular fibrillation with the front pad placed on the upper right torso above the right nipple just below the clavicle (small circle) and the lateral pad placed on the side of the chest below the left breast to avoid interfering with the surgical site (large circle). Note that the lateral pad should originally be placed in the 5th or 6th intercostal space on the left anterior axillary line. After discussion with the surgeon, it was decided that the lateral pad should be placed in this location to avoid the surgical site and ensure a sufficient sterile area.

For patients with CPVT, premedication such as with benzodiazepines helps reduce anxiety^2^; however, further pharmacologic anxiolysis was omitted because this patient was sufficiently reassured by the explanation the day before, and her anxiety was not significant on the day of surgery. After entering the operating room, she maintained an HR of 50 bpm and systolic blood pressure (BP) in the range of 90–100 mmHg. Standard monitoring was applied and included electrocardiography, noninvasive BP, and pulse oximetry. A bispectral index (BIS) sensor was attached to her forehead for anesthetic depth monitoring. In addition, defibrillator pads were applied in preparation for unexpected intraoperative VF. The front pad was placed on the upper right torso above the right nipple just below the clavicle, and the lateral pad was placed on the side of the chest below the left breast to avoid interfering with the surgical site and ensure a sufficient sterile area (Figure 2).

**FIGURE 3:**
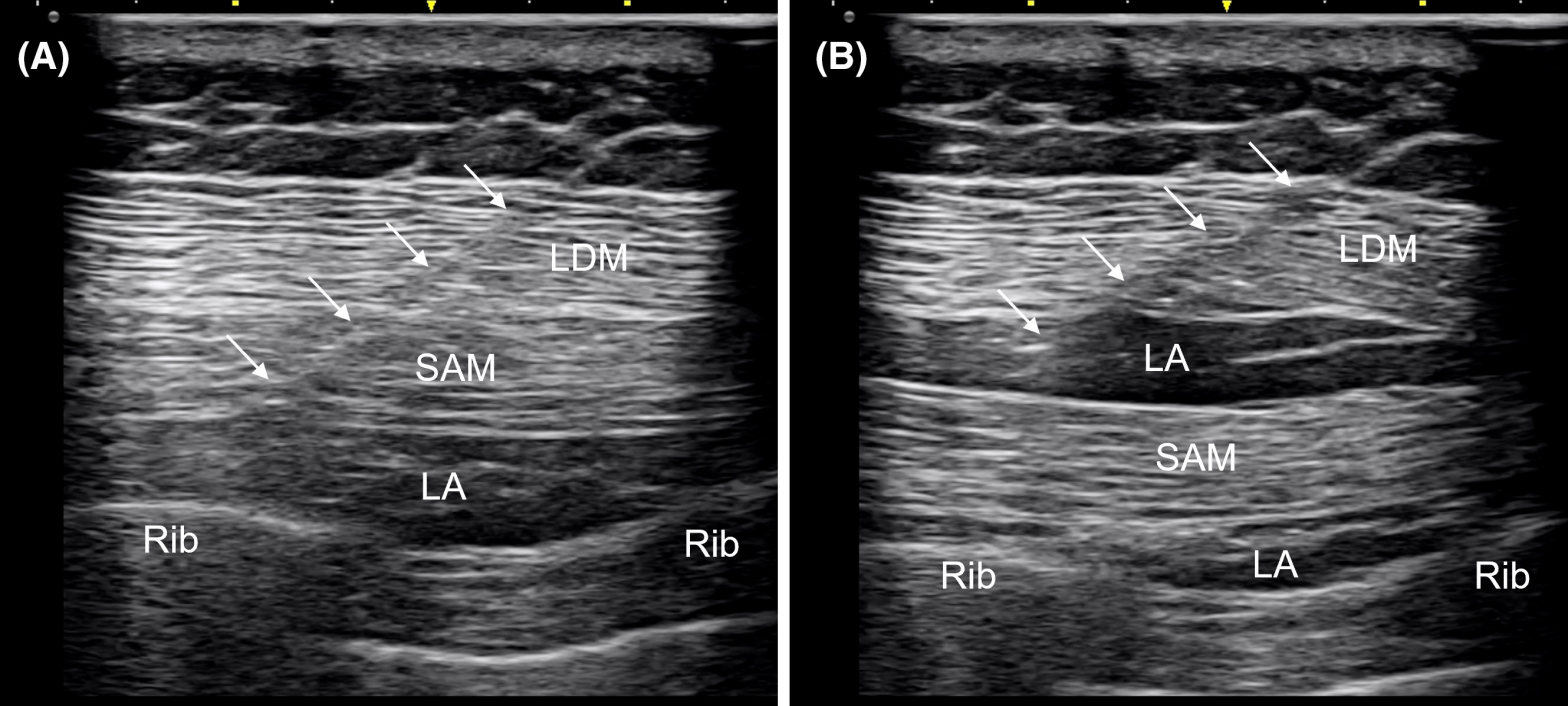
Ultrasound images of the serratus plane block. Local anesthesia is injected into the deep plane (A) and superficial plane (B) through a Tuohy needle (arrows). LA, local anesthetics, LDM, latissimus dorsi muscle, SAM, serratus anterior muscle.

Anesthetic induction and maintenance were performed with propofol and remifentanil by using two target‐controlled infusion pumps (Injectomat TIVA Agilia; Fresenius Kabi). To reduce propofol injection pain, anesthesia induction was initiated after injecting 40 mg of lidocaine intravenously. After confirming the patient's level of consciousness and administering an intubating dose of rocuronium 50 mg, an assistant anesthesiologist performed arterial catheterization on the radial artery to continuously monitor the BP. During general anesthesia, propofol was adjusted depending on BIS values, and remifentanil was adjusted depending on hemodynamic parameters. Smooth tracheal intubation was required to attenuate possible sympathetic stimulation; therefore, a silicone double‐lumen tube (DLT) (35 Fr; Human‐Broncho; Insung Medical Co.) was placed in the trachea by using a videolaryngoscope (C‐MAC; Karl Storz). Under videolaryngoscopic guidance, Cormack–Lehane grade 1 view was achieved, and tracheal intubation was easily completed. At that time, an HR of 50 bpm and systolic BP of 90 mmHg were maintained, with continuous infusion of remifentanil at an effect‐site concentration of 2 ng/mL. The proper DLT position was identified using a fiberoptic bronchoscope. After carefully changing the patient to the right lateral decubitus position, an ultrasound‐guided serratus plane block (SPB) was performed using ultrasound (SONIMAGE HS2; Konica Minolta) with a high‐frequency linear probe for perioperative pain control. After mixing 15 mL of 0.75% ropivacaine and 15 mL of normal saline to make 30 mL of 0.375% ropivacaine, 15 mL each was injected into the deep plane below the serratus anterior muscle, and the superficial plane above the serratus anterior muscle via a 22‐G Tuohy needle by using the in‐plane technique (Figure 3).

After the anesthesiologist performed the SPB, the surgeon disinfected the surgical site and covered it with surgical drapes. Left‐sided thoracic sympathectomy from T2 to T5 and resection of the lower half of the stellate ganglion were successfully conducted after performing single‐port video‐assisted thoracoscopic (VATS) exploration in the left third intercostal space. No arrhythmic events occurred during the procedure. With regard to the BP before the incision after anesthesia induction, the systolic BP was approximately 80 mmHg (HR, 60–70 bpm), and no hypotensive event occurred that required vasopressor use, except for administering 50 μg of phenylephrine twice. At the end of the surgery, 0.3 mg of ramosetron and 500 mg of paracetamol were injected intravenously. After changing the patient to a supine position and performing endotracheal suction sufficiently, two‐lung recruitment was conducted. After discontinuing propofol infusion, sugammadex 200 mg (4 mg/kg) was administered as the reversal agent of the muscle relaxant for rapid and reliable muscle tone recovery. To maintain body temperature during surgery, forced air warming was administered while covering the patient's arms and legs with a warm cloth, except for the surgical site. Forced air warming was similarly administered while covering the whole body with a warm cloth to maintain body temperature during the waking‐up process after the surgery. When the BIS rose to approximately 80, we tried to wake her by only calling her name or by light shoulder tapping. Extubation was performed because she responded to auditory stimuli such as calling her name and cooperatively responded to commands to encourage deep breathing. Immediately after extubation, she breathed spontaneously and smoothly without vigorous coughing. The BP was maintained at 120/70 mmHg and HR was maintained at 60–70 bpm with no abnormal findings on the electrocardiogram.

**FIGURE 4:**
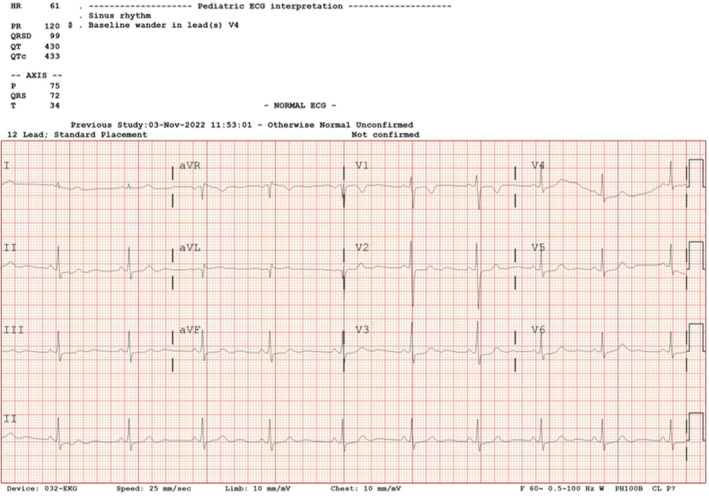
The follow‐up electrocardiogram, obtained 3 months after surgery, shows a sinus rhythm and a heart rate of 61 beats/min.

Vital signs were stable during the postanesthetic care unit (PACU) stay. The patient's mother was allowed into the PACU to provide psychological support. Immediately after being admitted to the PACU, the pain was 6 points on the numeric rating scale (NRS). We administered 30 μg of fentanyl to control pain. Dexamethasone 5 mg was administered because the patient complained of post‐extubation throat discomfort. The NRS was afterward checked as 2 points. She did not complain of discomfort; hence, she was transferred to the ward 30 min after the PACU admission. When she was examined after surgery, she had no symptoms of Horner syndrome. The pain score was checked at 6 h with a resting NRS of 2 and an active NRS of 3. At the first and second months after discharge, she was stable without any symptoms during an outpatient visit. The doses of flecainide and bisoprolol were gradually reduced. In the follow‐up electrocardiogram, conducted in the third month, the sinus rhythm HR was 61 bpm (Figure 4), and she is currently on outpatient follow‐up with no significant event identified. This paper was approved by the institutional review board of Kyungpook National University Chilgok Hospital in Daegu, Korea (KNUCH 2022‐11‐021). The patient and her parents provided informed consent for the publication of this case report.

## 3 DISCUSSION

CPVT mutations render the RyR2 calcium‐release channels hyperactive during sympathetic activation. The resulting DAD induces bigeminy of the right and left bundle branches, and has been proposed as the pathogenesis of life‐threatening bidirectional VT, the most characteristic feature of CPVT.^6^ The first clinical manifestation of CPVT is syncope or aborted sudden cardiac death,^1^, ^6^ which differs from seizures in that almost all syncopal events are associated with physical activity or emotional stress and do not occur at a resting state.^6^ Therefore, methods are needed to blunt the effect of catecholamines. Guidelines for treating patients with CPVT recommend various therapeutic options,^7^, ^8^ and patients diagnosed with CPVT require lifestyle changes that limit or avoid competitive sports or stressful environments.^7^, ^8^ In addition, beta‐adrenergic receptor blockers (BB) are recommended as the primary treatment for all symptomatic patients, and adding flecainide, a sodium‐channel blocker, to BB may be useful.^7^ The guideline recommends LCSD if patients are intolerant to or have a contraindication to this first‐line BB medication and if recurrent syncope or VT or several implantable cardioverter defibrillator (ICD) shocks occur, despite continued medication.^7^ With regard to ICD implantation, life‐long and routine device replacement is required in young patients and is associated with device malfunction, infection, and psychological problems.^9^, ^10^, ^11^, ^12^ Moreover, ICD shock is generally adequate for VF; however, it is ineffective for terminating polymorphic VT, bidirectional VT, and electrical storm, especially in patients with CPVT.^13^ Pain and fear related to ICD shock can increase catecholamine release, which can trigger an electrical storm.^11^ Thus, bridging to ICD implantation in small children is traditionally a major clinical indication for LCSD.^14^ LCSD interrupts localized norepinephrine release from the sympathetic nerve ending to the left ventricular myocardium by preganglionic denervation and transiently decreases cardiac tissue norepinephrine without a decrease in myocardial contractility and reduction in HR, thereby increasing VF threshold.^12^, ^15^, ^16^ Recent large multicenter studies in patients with LQTS and CPVT have also shown that LCSD effectively reduces cardiac events and prevents ICD discharges due to shock‐associated sympathetic surges.^11^

In the anesthetic management of CPVT patients undergoing LCSD, preventing sympathetic activation that triggers severe arrhythmia is vital; therefore, sufficient anesthesia and analgesia should be maintained. In particular, performing smooth anesthesia induction is crucial. For this purpose, methods to minimize the catecholaminergic response to laryngeal instrumentation, such as laryngoscopy and tracheal intubation, should be used. As one method, we performed DLT intubation by using a videolaryngoscope (VL). With regard to a VL, a camera at the distal end of the blade provides better glottis exposure, and minimal head and neck manipulation is possible.^17^ Therefore, some studies have shown that the force applied to the tongue and supraglottic tissue by the laryngoscope was small, which can reduce soft tissue trauma and sore throat incidence.^17^ A study comparing GlideScope and Macintosh direct laryngoscope (DL) in DLT intubation demonstrated that the incidence of sore throat and hoarseness and the severity of sore throat were significantly lower in the group using a VL.^18^ Additionally, we used a silicone DLT in the present patient. Silicone DLTs have a softer body and a more flexible bronchial tip than polyvinyl chloride (PVC) DLTs. Furthermore, a related study has shown that the incidence of postoperative sore throat at 1 h and 24 h and the intensity up to 24 h were significantly lower in patients using a silicone DLT rather than a PVC DLT.^19^ However, the supporting data regarding whether a VL or silicone DLT can attenuate the hemodynamic response are limited and controversial. In the aforementioned comparative study of VL‐versus DL‐guided DLT intubation, hemodynamic data did not show any significant difference between the two groups,^18^ and the silicone versus PVC DLT comparative study did not directly address these variables.^19^ However, methods to minimize a sore throat (i.e., pain in the pharynx and larynx) and the anxiety and emotional stress occurring because of a sore throat may eventually prevent arrhythmia provocation in patients with CPVT.

Furthermore, since LCSD is not a curative treatment^14^, ^16^ and smooth recovery from anesthesia is crucial even after surgery, adequate perioperative analgesia is essential. For adequate perioperative analgesia, we applied ultrasound‐guided SPB. This recently introduced technique for treating unilateral thoracic wall pain is relatively simple, easy, and safe to perform.^20^, ^21^, ^22^, ^23^ Under ultrasound guidance, when local anesthetics are injected into the plane above and below the serratus anterior muscle at the mid‐axillary line and fifth rib level, they spread along the plane and block the lateral branches of the intercostal nerve passing through. In addition, since the long thoracic and dorsal thoracic nerves pass through the serratus anterior muscle surface, the SPB effect can successfully cover the anterolateral chest wall, which is the VATS surgical incision site in most patients.^20^, ^21^ Moreover, it blocks only the peripheral nerve; however, it can reduce the sympathetic response, such as increased BP or HR, by blocking nociceptive afferent transmission.^22^ A recent study^22^ has shown no differences in intraoperative hemodynamic parameters in patients undergoing SPB, but intraoperative remifentanil consumption was significantly lower. In addition, a study^23^ has shown that the pain score and opioid consumption were significantly decreased during the early postoperative period in patients who underwent SPB, and the quality of recovery score was improved. Thoracic epidural analgesia (TEA) has been reported to be effective in patients with ventricular arrhythmia, including in patients with CPVT undergoing LCSD.^8^, ^24^ Both pain relief and sympathetic block effects can be obtained by injecting local anesthetics or opioids into the epidural space from T1 to T4. TEA is also beneficial in reducing postoperative arrhythmia, especially in cardiac surgery.^24^ In animal experiments, TEA has been shown to increase the threshold of VF during acute myocardial ischemia,^25^ increase ventricular repolarization and the effective refractory period,^26^ and reduce spatial heterogeneity in repolarization induced by sympathoexcitation,^27^ similar to the surgical cardiac denervation effect mentioned above. However, we thought that inserting a thoracic epidural catheter before anesthesia could cause the patient to feel anxious or complain of pain due to the procedure. In addition, considering the serious neurologic complications that may accompany neuraxial block when performed by clinicians unfamiliar with the procedure under general anesthesia,^28^ TEA was excluded from the options. However, a SPB, which can be safely performed with ultrasound guidance, even in the lateral decubitus position after anesthesia induction, may be suitable to achieve pain control and the consequent attenuation of sympathetic response in timorous adolescent patients. SPB has the advantage of minimizing systemic side effects caused by opioids such as postoperative nausea and vomiting (PONV) since it reduces opioid consumption.^22^, ^23^

As extubation itself can also be physically stressful, increasing BP and HR, consideration should be given to smooth extubation by preventing airway irritation and cough in addition to pain control for gentle recovery from anesthesia.^29^, ^30^, ^31^ Several options have been introduced for this, including deep extubation, exchanging the endotracheal tube to a laryngeal mask airway prior to extubation, and limiting unnecessary stimulation on emergence such as the “No‐Touch technique”.^30^, ^32^, ^33^ Pharmacologic interventions such as intravenous remifentanil, dexmedetomidine, or lidocaine can be added to facilitate these techniques.^30^, ^31^, ^34^, ^35^ Although these are commonly available medications, opioids and dexmedetomidine may worsen bradycardia and hypotension in patients with preexisting low HR and BP, and opioids can also cause PONV, which leads to significant stress.^31^ Additional intravenous lidocaine may cause local anesthetic overdose and systemic toxicity in combination with ropivacaine previously administered for SPB. Considering the patient's condition and each method's availability and pitfall, we only applied a stimulus‐limiting method, allowing only mild auditory or tactile stimuli to the patient during anesthetic emergence.

In addition to minimizing a patient's sympathetic response to external stimuli, clinicians should pay attention to avoiding drugs with intrinsic sympathomimetic activity that act on beta‐adrenergic receptors among the medications commonly used by anesthesiologists.^2^, ^8^ Most anesthetics are safe for use^8^, ^36^, ^37^; however, ketamine should be avoided because of its sympathomimetic properties.^2^, ^38^ In addition, among volatile agents, if the concentration of desflurane is rapidly increased, then sympathetic activation and an increase in catecholamine plasma concentration can be induced.^38^ Hence, anesthesiologists must take care. Most nondepolarizing neuromuscular blocking agents (NMBAs) and their reversal agents such as sugammadex have minor effects on the cardiac electrophysiology and can therefore be used safely.^2^, ^36^, ^38^ However, succinylcholine, a depolarizing NMBA, may cause sympathetic activation and increased catecholamine concentration and can make patients with electrolyte problems susceptible to arrhythmias.^38^ Thus, succinylcholine should be used cautiously when necessary. Moreover, beta‐adrenergic agonists should be avoided as a drug for treating hypotension during general anesthesia because they can cause fatal arrhythmias in patients with CPVT. Using phenylephrine, a pure alpha‐adrenergic agonist, is safe.^8^ Treating bradycardia, which causes hypotension, requires treatment with atropine or pacing without using beta‐agonist agents.^8^

Current treatment strategies for CPVT, such as pharmacotherapy, ICD, and LCSD, have improved outcomes but remain suboptimal and leave some patients at significant risk.^39^ Despite standard treatment, the long‐term incidence of life‐threatening events is still estimated to exceed 50% over 15 years of follow‐up.^39^ The prognosis is still not good, although it has improved in recent years compared to earlier reports of approximately 40% of patients dying within 10 years of diagnosis.^6^ Gene therapy has been researched as a new treatment for cure, and to date, all studies of gene therapy to treat CPVT have been preclinical studies using animal models and stem cell‐derived cardiomyocytes.^39^ As yet, not all sudden cardiac events in CPVT patients can be prevented, and the same in even those undergoing LCSD as our patient. Therefore, we should still recognize the several aforementioned methods of preventing sympathetic activation in the management of CPVT patients.

## 4 CONCLUSION

Sympathetic activation should be avoided in patients with CPVT to prevent the provocation of a fatal arrhythmia, even when performing LCSD, a surgical treatment method for patients with CPVT. Anesthetic management is needed to blunt the adrenaline effect due to various stimuli during the preoperative, intraoperative, and postoperative periods (i.e., during the perioperative period). Hence, tracheal intubation was performed using a VL with a silicone DLT, and ultrasound‐guided SPB was additionally applied in the present patient. Surgery and anesthesia were successfully performed without special arrhythmic events, and recovery was possible without adverse effects. Therefore, these simple and safe options can be useful methods for CPVT patients undergoing LCSD, although further studies on their effectiveness are needed.

## AUTHOR CONTRIBUTIONS

**Kyung‐Hwa Kwak:** Conceptualization; writing – original draft; writing – review and editing. **Young‐Woo Do:** Resources; writing – review and editing. **Taeyoung Yu:** Data curation; investigation; writing – review and editing. **Jinyoung Oh:** Supervision; writing – review and editing. **Sung‐Hye Byun:** Conceptualization; investigation; resources; visualization; writing – original draft; writing – review and editing.

## FUNDING INFORMATION

This study received no external funding.

## CONFLICT OF INTEREST STATEMENT

The authors have no conflict of interest to disclose.

## ETHICS STATEMENT

This paper was approved by the institutional review board of Kyungpook National University Chilgok Hospital in Daegu, Korea (approval no., KNUCH 2022–11‐021).

## CONSENT

Written informed consent was obtained from the patient and her parents for publication of this case.

## Data Availability

The data presented in this study are available on request from the corresponding author.
